# Prognostic value of long-term trajectories of depression for incident diabetes mellitus in patients with stable coronary heart disease

**DOI:** 10.1186/s12933-021-01298-3

**Published:** 2021-05-13

**Authors:** Raphael S. Peter, Andrea Jaensch, Ute Mons, Ben Schöttker, Roman Schmucker, Wolfgang Koenig, Hermann Brenner, Dietrich Rothenbacher

**Affiliations:** 1 Institute of Epidemiology and Medical Biometry, Ulm University, Ulm, Germany; 2 Division of Clinical Epidemiology and Ageing Research, German Cancer Research Center (DKFZ), Heidelberg, Germany; 3 Faculty of Medicine and University Hospital Cologne, Heart Center, University of Cologne, Cologne, Germany; 4 Network Ageing Research, University of Heidelberg, Heidelberg, Germany; 5 Klinik Schwabenland, Isny-Neutrauchburg, Germany; 6 Deutsches Herzzentrum München, Technische Universität München, Munich, Germany; 7 German Centre for Cardiovascular Research (DZHK), Partner Site Munich Heart Alliance, Munich, Germany

**Keywords:** Depression, Trajectories, Diabetes mellitus, Coronary heart disease

## Abstract

**Background:**

Diabetes mellitus (DM) and depression are bidirectionally interrelated. We recently identified long-term trajectories of depression symptom severity in individuals with coronary heart disease (CHD), which were associated with the risk for subsequent cardiovascular events (CVE). We now investigated the prognostic value of these trajectories of symptoms of depression with the risk of incident DM in patients with stable coronary heart disease.

**Methods:**

The KAROLA cohort included CHD patients participating in an in-patient rehabilitation program (years 1999/2000) and followed for up to 15 years. We included 1048 patients (mean age 59.4 years, 15% female) with information on prevalent DM at baseline and follow-up data. Cox proportional hazards models were used to model the risk for incident DM during follow-up by depression trajectory class adjusted for age, sex, education, smoking status, body mass index, and physical activity. In addition, we modeled the excess risk for subsequent CVE due to incident DM during follow-up for each of the depression trajectories.

**Results:**

DM was prevalent in 20.7% of patients at baseline. Over follow-up, 296 (28.2%) of patients had a subsequent CVE. During follow-up, 157 (15.0%) patients developed incident DM before experiencing a subsequent CVE. Patients following a high-stable depression symptom trajectory were at substantially higher risk of developing incident DM than patients following a low-stable depression symptom trajectory (hazard ratio (HR) = 2.50; 95% confidence interval (CI) (1.35, 4.65)). A moderate-stable and an increasing depression trajectory were associated with HRs of 1.48 (95%-CI (1.10, 1.98)) and 1.77 (95%-CI (1.00, 3.15)) for incident DM. In addition, patients in the high-stable depression trajectory class who developed incident DM during follow-up were at 6.5-fold risk (HR = 6.51; 95%-CI (2.77, 15.3)) of experiencing a subsequent cardiovascular event.

**Conclusions:**

In patients with CHD, following a trajectory of high stable symptoms of depression was associated with an increased risk of incident DM. Furthermore, incident DM in these patients was associated with a substantially increased risk of subsequent CVE. Identifying depressive symptoms and pertinent treatment offers might be an important and promising approach to enhance outcomes in patients with CHD, which should be followed up in further research and practice.

## Background

Diabetes mellitus (DM) is a global public health problem that increases strongly in all parts of the world [1]. Diabetes mellitus and depression are bidirectionally interrelated [2]. On the one hand, there is an increased risk of depression in patients with prevalent DM [3]. On the other hand, individuals diagnosed with depression or depressive symptoms are at increased risk of incident DM [4].

Among patients with DM, depression has been associated with a higher prevalence of microvascular and macrovascular complications [5–7]. In addition, symptoms of depression have been associated with poorer adherence to cardio-metabolic therapies in DM patients [8].

In addition, coronary heart disease (CHD) is still the leading cause of morbidity and mortality worldwide [9]. DM and CHD share common risk factors and some pathogenic pathways like chronic low-grade inflammation [10], a well-established marker in CHD pathology. As indicated by the serum level of C-reactive protein (CRP), systemic vascular inflammation may play multiple roles in the progression and destabilization of CHD and is also relevant in patients with already existing CHD [11]. Low-grade systemic inflammation seems to play also a role in the pathophysiology of depression [12].

We recently identified long-term trajectories of depression symptom severity in patients with coronary heart disease during long-term follow-up (CHD) [13], namely a low-stable, a moderate-stable, an increasing, and a high-stable trajectory class. The high stable depression class comprising about 3.3% of patients was associated with a substantially increased risk (hazard ratio = 2.47) of subsequent fatal or non-fatal cardiovascular events (CVE).

As there is evidence that depression is a risk factor for DM, trajectories of symptoms of depression may also be associated with incident DM during follow-up of CHD patients, and incident DM might again further increase the risk of subsequent CVE. However, results are inconsistent yet but might have important implications for provisions and timing of secondary prevention measures during in-patient rehabilitation and for long-term outpatient care, but also for further understanding of shared disease pathways of CHD and diabetes. To our knowledge, long-term trajectories of symptoms of depression in relation to DM in patients with CHD have not been investigated so far.

In this study, we investigated the prognostic value of long-term trajectories of symptoms of depression on the occurrence of incident DM in patients with CHD and the excess risk for subsequent CVE due to DM in patients following these different depression trajectories.

## Methods

### Subjects

The prospective KAROLA cohort study included patients with CHD (International Classification of Diseases (ICD), 9th Rev. pos. 410–414) aged 30–70 years participating in an in-patient rehabilitation program between January 1999 and May 2000 in one of two rehabilitation clinics in Germany (Schwabenland-Klinik, Isny, and Klinik am Südpark, Bad Nauheim), as previously described [13, 14]. In Germany, all patients discharged from an acute care hospital after an acute coronary syndrome or coronary artery bypass grafting are offered a comprehensive in-hospital rehabilitation program (on average about three weeks long). KAROLA only included patients admitted to in-patient rehabilitation within 3 months after their first acute event or coronary artery bypass grafting treated in an acute care hospital. Of all eligible patients admitted to the in-patient rehabilitation clinic during the recruitment period, 58 % (n = 1206) agreed to participate.

### Assessment of symptoms of depression and trajectory class membership

The German version [15] of the hospital anxiety and depression scale (HADS) was included in questionnaires at the 1-, 3-, 6-, 8-, and 15-year follow-up. HADS is a standardized, self-administered questionnaire containing 14 questions to quantify generalized anxiety and depression (seven items each) in medical patients [16] and performs well in patients with cardiac diseases. All items are scored on a four-point Likert scale (0–3 points). A summary score is calculated for the depression subscale ranging from 0 to 21. Only the depression subscale of the HADS is included in the present analysis.

We previously identified four long-term trajectories of depression symptoms within the KAROLA study population using a joint latent class mixture time-to-event model [13]. Based on this model, trajectory class membership probabilities were assigned to each individual in the current analysis dataset.

### Follow-up and evaluation of cardiovascular disease events and diabetes mellitus

Up to the 15-year follow-up (ending in May 2015), each patient and the actual primary care physicians were contacted regularly (at 1, 3, 6, 8, 10, 13 and 15 years) by mail and asked to complete standardized questionnaires regarding comorbidity, subsequent non-fatal CVEs, and medication and treatment since discharge from the in-patient rehabilitation clinic. The vital status during follow-up was assessed via the residents’ registration office. In case of death, the exact date and location of death were obtained, and the death certificate was obtained from the local Public Health department. The main cause of death was coded according to the International Classification of Diseases. A subsequent CVE was defined as cardiovascular disease (CVD) as the main cause of death (ICD-9 pos. 390–459; ICD-10 pos. I0-I99 and R57.0) or a primary care physician reporting a non-fatal myocardial infarction or stroke. Incident DM was defined as a primary care physician reporting a new DM diagnosis for the first time. Prevalent diabetes at baseline included DM reported by the primary care physician, by the patient or the patient reporting intake of antidiabetic medication.

### Statistical methods

Characteristics by previously identified depression symptom trajectories are presented as means and standard deviations weighted by class membership probabilities for continuous variables and as frequencies weighted by class membership probabilities for discrete variables.

The hazard ratios (HR) for incident diabetes by trajectory class were modeled as a Cox proportional hazards model with time since hospital admission as the underlying timescale. Time at risk started at discharge from the in-patient rehabilitation clinic (left truncation) and ended when reaching the DM endpoint. Time at risk was censored when the patient was lost to follow-up, the patient died, experienced a subsequent CVE, or reached the last (15-year) follow-up. Models were fitted on an extended dataset (one row per individual and trajectory class) and weighted by trajectory class membership probability. The HRs for the excess CVE risk due to DM were modeled using Cox models with DM as a time-varying exposure. We used the same timescale as for the previous models but with subsequent CVE as the endpoint, again on an extended dataset weighted by class trajectory membership probability. The models included a trajectory-class-membership (as strata) by DM interaction term.

We used two different adjustment sets, one including age and sex, and one additionally adjusted for education (< 10 years vs. ≥10 years), smoking status (former/current smoker vs. never smoker), body mass index (BMI), and physical activity (up to 3 h of sweaty activity per week, more than 3 h per week, or not answered). When incident DM was analysed, patients with prevalent DM at baseline were excluded. All confidence intervals for HRs were derived using the model-based robust variance estimates. All analyses were performed with R (R Foundation for Statistical Computing, Vienna, Austria) version 3.6.3.

## Results

**Fig. 1:**
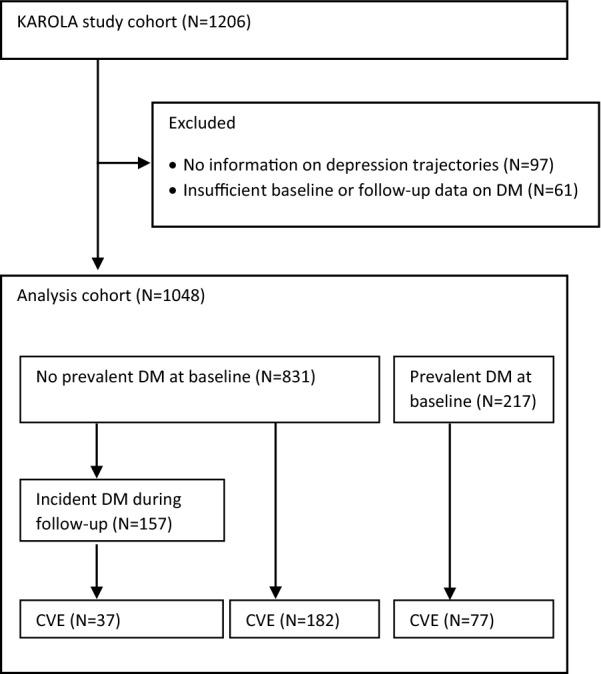
Study flow chart

Information on depression trajectories and DM was available for 1048 patients out of 1206 individuals who agreed to participate (Fig. 1).

These patients were on average 59.4 years old at study inclusion, only 15% of patients were female, and 20.7% already had prevalent DM at baseline (Table 1). Over 15 years of follow-up, 296 (28.2%) of patients had a subsequent CVE, 157 (15.0%) of the total study population developed incident DM before experiencing a subsequent CVE, leading to an incidence rate of 17.2 per 1000 patient-years.

**Table 1:** Main characteristics of the study population (all characteristics refer to baseline, except incidence figures)

		N = 1048
Age (years), mean (SD)		59.4 (8.0)
Female sex, n (%)		157 (15.0)
Education < 10 years, n (%)		627 (59.8)
Former/current smoker, n (%)		709 (67.6)
Body mass index (kg/m^2^), mean (SD)		26.9 (3.3)
Physical activity, n (%)^a^		
Up to 3 h per week		477 (45.5)
More than 3 h per week		544 (51.9)
Not answered		27 (2.6)
History of myocardial infarction, n (%)		604 (57.6)
Prevalent diabetes mellitus, n (%)		217 (20.7)
Clinical score (angiographic evaluation), n (%)		
0- or 1-vessel disease		274 (26.1)
2-vessel disease		280 (26.7)
3-vessel disease		445 (42.5)
Unknown		49 (4.7)
Subsequent cardiovascular (CV) events, N (%)		296 (28.2)
Incident diabetes (until subsequent CV event), N (%)		157 (15.0)

^a^ sweaty activities within the year prior to the index event

**Fig. 2:**
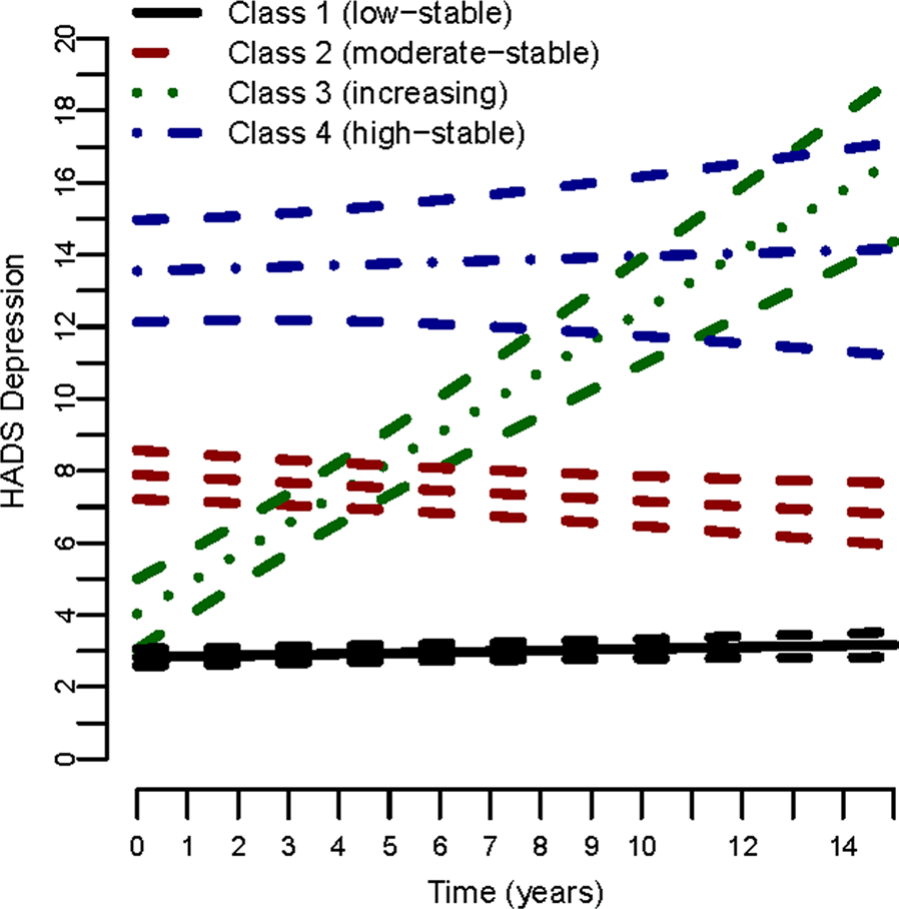
Previously identified depression symptom trajectories (means with 95% confidence intervals) according to HADS depression score at baseline and during follow-up

The previously identified depression trajectories of this cohort are shown in Fig. 2. Patients with a high probability of belonging to the high stable depression trajectory were more likely to have prevalent DM already at baseline (Table 2) (30.1% for the high-stable class vs. 19.1% for the low-stable class). Also, subsequent CVEs were much more common in the high-stable class (47.4% vs. 26.3% for the low-stable class), as was incident DM (21.9% vs. 13.5% for the low-stable class).

**Table 2:** Characteristics by previously identified depression symptom trajectories

	Class 1 (low-stable)^a^	Class 2 (moderate-stable)^a^	Class 3 (increasing)^a^	Class 4 (high-stable)^a^
N (%)	711.5 (67.9)	245.5 (23.4)	56.7 (5.4)	34.3 (3.3)
Age (years), mean (std)	59.6 (7.9)	58.6 (8.0)	61.3 (8.4)	57.3 (8.2)
Female sex, N (%)	102.3 (14.4)	41.4 (16.9)	8.2 (14.4)	5.1 (14.8)
Prevalent diabetes mellitus at baseline (%)	163.2 (19.1)	57.0 (23.2)	13.5 (23.7)	10.3 (30.1)
Subsequent cardiovascular (CV) events, N (%)	187.2 (26.3)	67.9 (27.7)	24.6 (43.4)	16.3 (47.4)
Incident diabetes (until subsequent CV event)^b^, N (%)	95.8 (13.5)	44.8 (18.3)	8.9 (15.7)	7.5 (21.9)

^a^ Statistics (including Ns) are weighted by the probability of class membership; Ns might therefore be non-natural numbers

^b^ Excluding patients with prevalent DM at baseline

The risk for incident DM was 2.83 times as high for the high-stable depression class as for the low-stable trajectory (Table 3), after adjustment for age and sex. Also, individuals following the moderate-stable and increasing depression trajectories faced a substantially increased risk (HRs of 1.55 and 2.50) for incident DM. Further adjustment for education, smoking status, BMI, and physical activity only slightly attenuated the HRs.

**Table 3:** Hazard ratio for development of DM during follow-up by depression trajectory class (N_Total_=831, N_Events_=157)

	N_Events_c	Follow-up (Person-years)^c^	Age- & sex-adjusted Hazard ratio (95 %-CI)^a^	Fully adjusted Hazard ratio (95 %-CI)^a,b^
Class 1 (low-stable)	95.8	5911	(ref.) 1.00	(ref.) 1.00
Class 2 (moderate-stable)	44.8	1783	1.55 (1.16, 2.08)	1.48 (1.10, 1.98)
Class 3 (increasing)	8.9	317	1.94 (1.10, 3.44)	1.77 (1.00, 3.15)
Class 4 (high-stable)	7.5	181	2.83 (1.53, 5.23)	2.50 (1.35, 4.65)

^a^ Excluding patients with prevalent DM at baseline

^b^ Adjusted for age, sex, education, smoking status, BMI, and physical activity

^c^ Statistics are weighted by the probability of class membership; Ns might therefore be non-natural numbers

Table 4 displays excess cardiovascular event (CVE) risk due to prevalent diabetes mellitus (DM) at baseline and incident DM during follow-up. When looking on the risk estimates of subsequent CVEs by DM prevalence at baseline vs. no prevalent DM, we found an increased risk for the low-stable, moderate-stable, and high-stable depression trajectory class. However, confidence intervals were wide and mostly included the null effect value. Interestingly, patients who fell into the high-stable depression trajectory class and who developed incident DM during follow-up were at 6.5-fold risk of experiencing a subsequent CVE compared to those in the same depression trajectory class who did not develop DM.

**Table 4:** Excess cardiovascular event (CVE) risk due to prevalent diabetes mellitus (DM) at baseline and incident DM during follow-up

	Hazard ratio (95 %-CI)		
	Prevalent DM vs. no prevalent DM^a^	Prevalent + incident DM vs. no DM	Incident DM vs. no DM^b^
N_CVE_/N_Total_	296/1048	296/1048	219/831
Age- and sex-adjusted models			
Class 1 (low-stable)	1.40 (1.03, 1.90)	1.39 (1.05, 1.85)	1.31 (0.82, 2.11)
Class 2 (moderate-stable)	1.51 (0.97, 2.36)	1.18 (0.79, 1.78)	0.76 (0.41, 1.39)
Class 3 (increasing)	0.66 (0.31, 1.42)	0.71 (0.38, 1.33)	0.80 (0.32, 1.97)
Class 4 (high-stable)	1.68 (0.75, 3.80)	4.65 (1.95, 11.1)	6.59 (2.69, 16.1)
Fully adjusted models^c^			
Class 1 (low-stable)	1.31 (0.95, 1.79)	1.31 (0.98, 1.75)	1.26 (0.77, 2.05)
Class 2 (moderate-stable)	1.45 (0.69, 2.27)	1.11 (0.74, 1.67)	0.72 (0.39, 1.33)
Class 3 (increasing)	0.66 (0.31, 1.40)	0.69 (0.37, 1.29)	0.78 (0.32, 1.86)
Class 4 (high-stable)	1.73 (0.78, 3.86)	4.66 (1.98, 10.9)	6.51 (2.77, 15.3)

^a^ Ignoring incident DM during follow-up

^b^ Excluding patients with prevalent DM at baseline

^c^ Adjusted for age, sex, education, smoking status, BMI, and physical activity

## Discussion

In this long-term cohort study, including over 1000 patients with stable CHD at baseline, we found that patients on a trajectory of high stable depression symptoms during long-term follow-up faced an increased risk of developing DM. In addition, those following a trajectory of high-stable depression symptoms who newly developed DM faced a substantial 6.5-fold increased risk for subsequent CVEs. Therefore, more attention should be given to symptoms of depression both in the initial phase of diagnosis of CHD during in-patient rehabilitation and also during long-term care of CVD-patients. If the diagnosis is confirmed by a clinical examination (as HADS is basically a screening instrument), a respective treatment of depression should be initiated. In addition, given the increased risk for DM, the metabolic state should be watched carefully to allow early preventive means.

To our knowledge, long-term trajectories of symptoms of depression in relation to DM in patients with CHD have not been investigated so far. However, depressive symptoms have previously been shown to be associated with DM incidence [17], and depression has been consistently associated with a higher prevalence of macrovascular complications in patients with DM [5–7]. Notably, patients with DM requiring glucose-lowering therapy and patients without diabetes but a prior myocardial infarction carry the same cardiovascular risk [18]. In addition, depression in CHD patients leads to lower adherence to oral hypoglycemic, antihypertensive, and lipid-lowering medication and unfavorable lifestyle choices [19, 20]. Therefore, it is not surprising that patients with a previous CVE and DM are at especially high risk for a subsequent CVE.

In this cohort of patients with CHD, we previously observed an increased risk for subsequent CVE in patients following a high-stable depression symptoms trajectory [13]. A finding now substantiated by the observation that also the risk for incident DM is increased, which subsequently increases the risk for CVE massively.

### Possible pathogenic mechanism linking depression, diabetes, and CHD

Solid evidence accumulates that depression is a risk factor for DM, irrespective of measures used to evaluate it, as seen in a recent meta-analysis [21]. Recent results of the Maastricht study indicate hyperglycemia itself may be involved in the etiology of depression [22]. In that study, several indicators of hyperglycemia (fasting plasma glucose, two-hour post-load glucose, and HbA1c) were consistently associated with a higher risk of incident depression independent of lifestyle factors. In addition, diabetes-associated microvascular dysfunction may be associated with the risk of incident depression [23].

DM is associated with chronic low-grade inflammation [24], as is depression [25–28]. Also, anti-cytokine treatments exhibit antidepressant effects in chronic inflammatory conditions [29], pointing to a causal role of chronic inflammation in the etiology of depression. Besides non-modifiable risk factors such as ethnicity, family history, and older age, the following modifiable risk factors play a role in DM etiology: obesity, physical inactivity, smoking, and unhealthy diet [30]. Especially obesity, but also physical inactivity and smoking may result in changes in inflammatory status. In obesity, dyslipidemia and changes in circulating leptin serum values may also be present. In a recent study from Augsburg, Germany, investigating the relevance of biomarker-defined pathways for the development of DM, and also for coronary heart disease, many associations, such as cytokines, endothelial dysfunction, hemostasis, hormone regulation, tissue remodeling, and others, showed similar strength for DM and CHD, but the insulin-like growth factor binding protein 2 explained DM risk best [10]. Interestingly, the risk associated with N-terminal prohormone of brain natriuretic peptide (NT-proBNP) for DM was inverse, whereas it was positive for CHD. This finding was substantiated in a subsequent analysis. High plasma concentrations of mid-regional pro atrial natriuretic peptide (MR-proANP), another peptide from the natriuretic peptide system, were also associated with a lower risk of incident DM and insulin resistance [31].

An earlier analysis of our data suggested that well-established risk factors like BMI, physical activity, or low-grade chronic inflammation might be associated with categories of symptoms of depression [32]. An inverse association between physical activity and depression symptoms was also described in the British Whitehall II study [33]. Physical activity is also clearly in associated with reduced risk of incident DM [34]. In our current analysis, the associations of depression symptom trajectories with incident DM were slightly attenuated by adjusting for baseline lifestyle factors (including BMI and physical activity).

Patients with depression seem to have an increased autonomic nervous system activity, increased hypothalamic-pituitary-adrenocortical axis (HPA) activity and associated cardiovascular adverse effects, including vasoconstriction, increased heart rate, and heart rate variability [4, 35]. Therefore, the increased activity of the HPA and the dysregulation of the immune system are discussed as risk factors for DM but may also play an important role in subsequent cardiovascular adverse effects.

## Strengths and limitations

As the study population is predominantly male, we cannot be sure that all findings equally apply to women as well. Furthermore, the study population was recruited about three weeks after the initial acute event and only included patients referred and willing to participate in an in-patient cardiac rehabilitation. Although cardiac rehabilitation is the standard treatment of CHD in Germany, not all patients participate in the program, which could lead to an underrepresentation of severely ill patients. However, in Germany, in general, over 80% of patients participated in cardiac rehabilitation after myocardial infarction at the time of baseline recruitment of this study [36]. In addition, a recent analysis of electronic medical records from the US, including patients with ischemic heart disease, showed that the presence of comorbid depression was associated with greater participation in cardiac rehabilitation [37]. While our analyses do not allow a causal interpretation, depression symptoms in CHD patients may still be used as risk markers for DM and an increased risk for subsequent CVE.

### Clinical implications

There is a need to decrease DM risk in patients with CHD, especially as those who have both diseases have a two- to six-fold increased risk for cardiovascular death [30]. As the HADS is basically a screening instrument, further psychiatric referrals or clinical assessments are still needed to confirm the diagnosis. If the diagnosis is corroborated, a cardiac rehabilitation program should include psychological and physical activity interventions. A comprehensive cardiac rehabilitation program including diet, physical activity, and stress management reduced cardiovascular mortality, non-fatal myocardial infarction, and stroke by 33% at 3 years [38]. In addition, a meta-analysis of six types of interventions in CHD patients found that psychological and behavioral interventions reduced depression scores, all-cause mortality, and incidence of non-fatal myocardial infarction [39]. While cardiac rehabilitation programs with psychological and physical activity interventions effectively reduce depression symptoms and improve prognosis [38, 39], the implementation of these interventions in cardiac rehabilitation programs needs further evaluation.

## Conclusions

In patients with CHD, following a trajectory of high stable symptoms of depression was associated with an increased risk of incident DM. Furthermore, incident DM in these patients was associated with a substantially increased risk of subsequent CVE. Identifying depressive symptoms and pertinent treatment offers might be an important and promising approach to enhance outcomes in patients with CHD, which should be followed up in further research and practice.

## Data Availability

The datasets used and/or analysed during the current study are available from the corresponding author on reasonable request.

## Abbreviations

BMI: Body mass index
CHD: Coronary heart disease
CI: Confidence interval
CRP: C-reactive protein
CVE: Cardiovascular event
DM: Diabetes mellitu
HADS: Hospital anxiety and depression scale
HR: Hazard ratio
ICD: International Classification of Diseases
MR-proANP: Mid-regional pro atrial natriuretic peptide
NT-proBNP: N-terminal prohormone of brain natriuretic peptide
